# AptaTrans: a deep neural network for predicting aptamer-protein interaction using pretrained encoders

**DOI:** 10.1186/s12859-023-05577-6

**Published:** 2023-11-27

**Authors:** Incheol Shin, Keumseok Kang, Juseong Kim, Sanghun Sel, Jeonghoon Choi, Jae-Wook Lee, Ho Young Kang, Giltae Song

**Affiliations:** 1 Division of Artificial Intelligence, Pusan National University, Busan, Republic of Korea; 2 Research & Development, NuclixBio Seoul, Republic of Korea; 3 School of Computer Science and Engineering, Pusan National University, Busan, Republic of Korea; 4 Center for Artificial Intelligence Research, Pusan National University, Busan, Republic of Korea

**Keywords:** Aptamer protein interaction, Transformer, Pretraing, Structural representation, SELEX

## Abstract

**Background:**

Aptamers, which are biomaterials comprised of single-stranded DNA/RNA that form tertiary structures, have significant potential as next-generation materials, particularly for drug discovery. The systematic evolution of ligands by exponential enrichment (SELEX) method is a critical in vitro technique employed to identify aptamers that bind specifically to target proteins. While advanced SELEX-based methods such as Cell- and HT-SELEX are available, they often encounter issues such as extended time consumption and suboptimal accuracy. Several In silico aptamer discovery methods have been proposed to address these challenges. These methods are specifically designed to predict aptamer-protein interaction (API) using benchmark datasets. However, these methods often fail to consider the physicochemical interactions between aptamers and proteins within tertiary structures.

**Results:**

In this study, we propose AptaTrans, a pipeline for predicting API using deep learning techniques. AptaTrans uses transformer-based encoders to handle aptamer and protein sequences at the monomer level. Furthermore, pretrained encoders are utilized for the structural representation. After validation with a benchmark dataset, AptaTrans has been integrated into a comprehensive toolset. This pipeline synergistically combines with Apta-MCTS, a generative algorithm for recommending aptamer candidates.

**Conclusion:**

The results show that AptaTrans outperforms existing models for predicting API, and the efficacy of the AptaTrans pipeline has been confirmed through various experimental tools. We expect AptaTrans will enhance the cost-effectiveness and efficiency of SELEX in drug discovery. The source code and benchmark dataset for AptaTrans are available at https://github.com/pnumlb/AptaTrans.

## Background

The scientific community focuses aptamers, which are biomaterials composed of chemically synthesized single-stranded DNA/RNA, because of their unique properties. Aptamers have emerged as frontrunners in the race to develop “next-generation biomaterials” owing to their high specificity and affinity for a wide range of target molecules, including viruses and proteins [1, 2]. Aptamers have a wide range of potential applications, particularly in diagnostics and therapeutics [3, 4].

One unique advantage of aptamers over other drugs such as antibodies is their distinctive qualities. Aptamers can identify and bind to molecular targets while larger drugs, such as antibodies, struggle with interacting effectively because they form smaller tertiary structures [1]. Additionally, aptamers demonstrate high stability under diverse conditions, resulting in a longer lifespan than many other drugs [5]. Furthermore, the low immunogenicity and toxicity profiles [6] of aptamers make them a viable choice for long-term therapeutic applications. Another important aptamer advantage is their easy production from a manufacturing perspective. The polymerase chain reaction can be used to manufacture large volumes of aptamers of excellent purity. In addition, the chemical synthesis of aptamers offers cost and time benefits compared with biological production methods [7]. Various factors contribute to the growing belief that aptamers can surpass antibodies in diagnostic and therapeutic applications [8].

The conventional technique for discovering aptamers, called the systematic evolution of ligands by exponential enrichment (SELEX), effectively isolates potential aptamers [9]. The SELEX technique consists of five primary stages: library generation, binding, separation, amplification, and replication. An extensive library of random sequences, typically comprised of DNA or RNA molecules, is created to act as a pool of potential aptamer candidates. From this library, random aptamer candidates are selected based on their capacity to bind to the target protein. The chosen aptamer candidates are then amplified and replicated using PCR or reverse transcription methods. In SELEX, each iteration of the selection and separation process, coupled with amplification and replication, is commonly termed a 'round.' Researchers typically conduct multiple rounds of SELEX, typically ranging from 5 to 20 rounds. However, it is important to note that each round of experimentation is time-consuming, often spanning several weeks to a few months. Additionally, SELEX success rates can be modest, leading to the synthesis of only a limited number of candidate aptamers for subsequent affinity characterization [10, 11].

Recent advancements in machine learning techniques have introduced new avenues for aptamer selection. Computational methods, specifically in silico approaches, have been developed to enhance the aptamer selection process [12]. Furthermore, deep learning models have demonstrated remarkable performance even in scenarios where limited data are available for protein binding prediction. For instance, in iHBP-DeepSSM [13], only 2460 data points were used to train a deep neural network (DNN) model for hormone-protein binding prediction. Similarly, Deep-AntiFP [14] and cACP-DeepGRAM [15] employed datasets of 2336 and 4475 data points, respectively, to predict antifungal and anticancer peptides using DNN models. These computational methods utilize data generated by SELEX and demonstrate potential in reducing both the time and costs associated with discovering aptamers. RaptRanker [16] is also a method that uses local sequence motifs and structural information for selects candidate aptamer sequences based on sequence frequencies. Additionally, other studies have aimed to predict API using specific aptamer and protein sequences. For example, Li et al. [17] devised a model using pseudo-amino acid composition to predict API. Various machine learning-based prediction models [18–20], employing techniques such as k-nearest neighbor, support vector machine, and ensemble methods, in addition to deep learning approaches [21, 22], have demonstrated high quality in predicting API. Despite these improvements, most current machine learning approaches often overlook that interactions between aptamers and proteins occur at the residue-structure level. These methods rely on deriving molecular characteristics from sequences, resulting in models that predict APIs based only on correlations in sequence configurations.

To address this issue, we propose AptaTrans, a deep learning framework for calculating the interaction matrix between aptamers and proteins at the monomer level. A transformer-based encoder is employed for sequence embedding and API prediction. To ensure optimal sequence embeddings, we pretrain the encoder using self-supervised learning strategies that utilize the predictions of masked tokens and the secondary structures of the molecules [23, 24]. We evaluated the effectiveness of the AptaTrans model using standard benchmark datasets commonly used for API prediction. The model exhibited a superior performance compared with existing data mining and machine learning methods. Additionally, we develop the AptaTrans pipeline, an integrated framework that combines the predictive abilities of AptaTrans with Apta-MCTS [25] to generate the candidate aptamer sequences. We validated our pipeline’s impact by using tools such as the ZDOCK Server [26] for scoring interaction. This study provides insights and tools for the discovery and development of aptamers, thereby facilitating their broader applications in diagnostics and therapeutics.

## Methods

### Data preparation

We collected a dataset widely used for API prediction [27, 28]. The dataset was obtained from the experimental results of aptamer-protein complex data, including both DNA and RNA aptamer-protein complexes [29]. We constructed the RNA benchmark datasets as conducted in Li et al. [17] to evaluate the performance in the same environment. Based on studies about the conversion of DNA and RNA [30, 31], for DNA aptamer-protein complexes, DNA sequences were converted into RNA sequences by substituting thymine (T) with uracil (U). The dataset was partitioned into a training set comprising 580 positive RNA aptamer-protein and 1,740 negative pairs, and a test set with 145 positives and 435 negatives for the training and evaluation of the API prediction model, as shown in Table 1.

**Table 1:** Benchmark dataset used for training and evaluating API prediction models

Number of positive pairs	Number of negative pairs	Description
580	1740	Train dataset
145	435	Test dataset

For pretraining our model, we used 166,136 protein sequences from the Protein Data Bank (PDB) [32]. We also collected 79,890 RNA sequences in the 'bpRNA-1 m' RNA dataset from bpRNA [33]. The 'bpRNA-1 m' dataset comprises over one million RNA sequences sourced from seven different platforms, including the PDB and Aptamer Base [29]. Accurate identification of a protein's secondary structure can serve as the basis for predicting many of the essential structural features required for 3D structure prediction. These secondary structures provide valuable insights into the functionality of the protein with other biomolecules. To generate protein secondary structure data that mirrors RNA secondary structures, we obtained protein data based on their tertiary structural information in the mmCIF/PDBx format from the PDB [34, 35]. A comprehensive distribution of both protein and RNA secondary structures is presented in Table 2.

**Table 2:** Distribution of protein and RNA secondary structures for pretraining

Types	PDB [32]	Types	bpRNA [33]
$\alpha$-helix	32.74%	Stem	48.50%
$\beta$-sheet	21.11%	Hairpin loop	22.51%
Turn	11.06%	Multi-loop	4.86%
$\beta$-bridge	1.22%	Internal loop	7.51%
$3_{10}$ helix	3.63%	Bulge	1.95%
Bend	9.15%	External loop	11.34%
Coil	20.45%	Pseudoknot	3.33%
$\pi$-helix	0.64%		

### Sequence tokenization using the k-mer and frequent consecutive subsequence (fcs) mining algorithms

We tokenized RNA and protein sequences to enable the exchange of information between tokens. Based on studies about the conversion of DNA and RNA [34, 35], for DNA aptamer-protein complexes, DNA sequences were converted into RNA sequences by substituting thymine (T) with uracil (U) per the RNA. The RNA sequences were tokenized using the k-mer algorithm, and the protein sequences were tokenized using the Frequent Consecutive Subsequence (FCS) mining algorithm [36, 37].

**Fig. 1:**
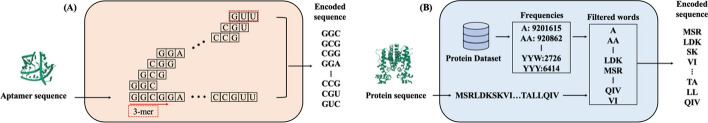
Sequence tokenization using two algorithms**.** (A) k-mer algorithm for aptamer sequences. (B) FCS mining algorithm for protein sequences

The k-mer algorithm divides a nucleotide or amino acid sequence into subsequences of length k, called words. For example, if k is three, as shown in Fig. 1A, sequence GGCGGAGAA…AACCGUC is divided into these substrings: GGC, GCG, CGG, GGA, …, CCG, CGU, and GUC.

The FCS mining algorithm is a type of WordPiece algorithm [38]. The algorithm can detect frequent consecutive substrings and generate a vocabulary that can be used for tokenization purposes. To detect these frequent consecutive substrings in the protein sequences, an initial vocabulary was constructed by including all possible substrings up to a length of three. We calculated the frequency of the most common sub-sequences for each protein sequence dataset. We excluded subsequence words with frequencies below the average from the initial vocabulary, retaining only the frequent subsequence words. In this study, we calculated the subsequent frequencies using the PDB datasets and generated a vocabulary through FCS mining. Sequences were hierarchically tokenized using this vocabulary. For example, if the vocabulary does not contain the MVS sequence and contains only MV and S tokens, the sequence is tokenized as {MV, S}. If the vocabulary includes all three tokens MV, S, and MVS, we represent sequence MVS as a single token referred to as {MVS}. For example, consider sequence MSRLDKSKVI…TALLQIV, which is shown in Fig. 1B. The filtered vocabulary represents this sequence as a set of tokens, including {MSR, LDK, SK, VI, …, TA, LL, QIV}.

### Building an API prediction model using transformer-based encoders

**Fig. 2:**
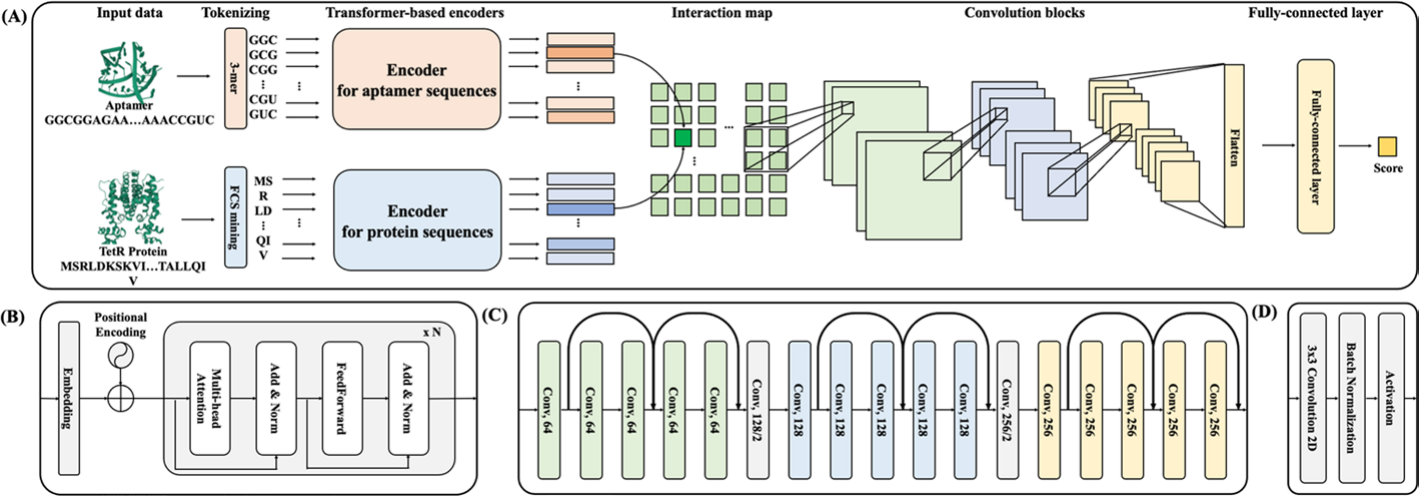
Architecture overview of the proposed model, AptaTrans. **A** The AptaTrans architecture consists of four parts: tokenization, transformer-based encoders, convolution blocks, and a fully connected layer. In (A), an interaction matrix is generated by computing the dot products of the pairs between the RNA and amino acid token embedding vectors from the encoders. **B** Shows a transformer-based encoder that includes an embedding layer, a positional encoder, a vanilla transformer encoder, **C** convolution layers, and **D** a single convolution layer that includes batch normalization and an activation function

After tokenizing the RNA and protein sequences, we built AptaTrans, a model that leverages transformer encoders to predict API at the monomer level [39, 40]. The conceptualization of AptaTrans was influenced by the Interactive Inference Network (IIN), a specialized neural network used to extract semantic features from the interaction domain to effectively interpret paired sentences [41]. MolTrans demonstrates the IIN's utility [37], which is employed to predict drug-target interactions. The AptaTrans model employs the architecture shown in Fig. 2A. AptaTrans utilizes tokenization algorithms, two encoders to represent the aptamer and protein sequences, convolution layers to extract information from the feature map (interaction map), and a fully connected layer to predict binding scores.

The model uses two primary inputs: an aptamer sequence ($x_{apta}$) and protein sequence ($x_{prot}$). Tokenization algorithms are applied to these sequences, followed by mining for 3-mers and frequent contiguous substrings (FCS) to obtain $x^{\prime}_{apta}$ and $x^{\prime}_{prot}$, respectively.

1 $$x^{\prime}_{apta} = {\text{3 - mer}}(x_{apta} ){, }x^{\prime}_{prot} = {\text{FCS(}}x_{prot} )$$

AptaTrans, uses a transformer encoder architecture, which is known for its ability to transform sequences into contextual vectors [39]. AptaTrans includes two distinct encoders: ${\text{Encoder}}_{prot} ( \cdot )$ for protein sequence $x^{\prime}_{prot}$ and ${\text{Encoder}}_{apta} ( \cdot )$ for aptamer sequence $x^{\prime}_{apta}$. As shown in Fig. 2B, each encoder consists of four major components: an embedding layer, positional encoding, a multi-head attention layer, and a feedforward layer. The embedding layer converts categorical word values into numerical vector representations, known as embedding vectors. Positional encoding was implemented to incorporate the positional relationships between words in the embedding vectors. The transformer architecture comprises numerous self-attention layers in parallel, and the multi-head attention layer [39] is a crucial component. Using this structure, encoders can capture a broad spectrum of contextual relationships among the token-embedding vectors. The multi-head attention layer is a valuable tool in molecular biology for capturing the interactions between monomers that define molecular structures. With these integrated components, our encoders adeptly process tokens $x^{\prime}_{prot}$ and $x^{\prime}_{apta}$, transforming them into enriched contextual representations denoted as $\tilde{E}_{prot}$ and $\tilde{E}_{apta}$. This operation translates sequences into an embedded space, thereby producing contextually aware representations. The encoder process can be described as follows:

2 $$\tilde{E}_{apta} = {\text{Encoder}}_{apta} (x^{\prime}_{apta} ){, }\tilde{E}_{prot} = {\text{Encoder}}_{prot} (x^{\prime}_{prot} )$$

AptaTrans creates an interaction matrix ($IM$) using contextualized embedding vectors: $\tilde{E}_{prot}$ and $\tilde{E}_{apta}$. The interaction value for each nucleotide-amino acid token pair is obtained using the dot product of the embedded token pairs as the aggregation function. This matrix contains interaction values representing the interactions between the nucleotide 3-mer tokens of the aptamer and amino acid sub-tokens of the protein. Each interaction value represents the strength of the corresponding interaction. The interaction matrix containing these interaction values is considered a feature map in the downstream layers.

3 $$IM = \tilde{E}_{apta} \cdot \tilde{E}_{prot}$$

AptaTrans employs specialized convolutional blocks to extract detailed information from the $IM$. The convolution blocks in AptaTrans capture both local and hierarchical features from the feature map. The sizes of these blocks were defined in three dimensions. Each dimension has five sublayers. The model includes two downsizing layers because it changes between these dimensions. The architecture consists of a set of blocks containing 17 layers. Each convolution block comprises a convolution layer, batch normalization, and an activation function, as shown in Fig. 2D. Our model utilizes a Gaussian Error Linear Unit (GELU) as an activation function [42]. The standard Gaussian cumulative distribution function multiplies the function's input. Because of its nonlinearity, this activation function provides advantages for backpropagation and is smoother than the commonly used rectified linear unit activation function. Figure 2C shows that AptaTrans comprises convolution blocks of three different sizes: 64, 128, and 256. Two residual connections are used for each size. Furthermore, a downsizing convolution layer is utilized during the transition between sizes. The single convolution blocks and convolution blocks utilized in AptaTrans are as follows:

4 $${\text{ConvBlock}}( \cdot ) = {\text{GELU(BatchNorm(Conv2d(}} \cdot {)))}$$

5 $$O = {\text{ConvBlocks(}}IM)$$

The output$\mathrm{O}$, from the convolution blocks is flattened and passed as input to the fully connected layer to obtain a prediction score that indicates whether binding has occurred. The final fully connected layer is defined as:

6 $$Score_{bind} = {\text{FullyConnected(Flatten(}}O))$$

### Pretraining encoders with self-supervised learning using masked tokens and secondary structures of molecules

To enhance the encoders in AptaTrans, we pretrained two encoders, ${\text{Encoder}}_{apta} ( \cdot )$ and ${\text{Encoder}}_{prot} ( \cdot )$ before training the API prediction model. Pretraining is a technique in which a model is trained on a large, general-purpose dataset before being finetuned for a primary, specific task. This approach enables the model to learn general features and patterns from the data. Self-supervised learning is a training technique in which a model learns from a dataset without explicit labels, and is trained to predict a correlated output using only the input data. This study utilized self-supervised learning with two pretraining tasks in AptaTrans: masked token prediction (MTP) and secondary structure prediction (SSP) [23, 24].

**Fig. 3:**
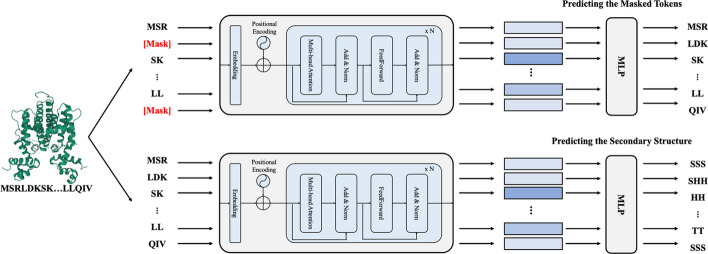
Example of pretraining techniques with two encoders. One is for the masked tokens prediction (top) and another for the secondary structure prediction (bottom)

The first task, masked token prediction (MTP), is similar to masked language modeling, which is a self-supervised learning technique commonly used in natural language processing for predicting masked or missing tokens in input text [24]. This technique aims to estimate the original masked tokens by considering the contextual information provided by the surrounding tokens. Consequently, the model learns to understand the relationships and context between the tokens in the input data. For example, in a protein sequence such as {MSR, LDK, SK, …, LL, QIV} that requires masking, the respective positions are replaced with {MSR, [mask], SK, …, LL, [mask]}, as illustrated in Fig. 3 (top). Nonetheless, unlike protein sequences, aptamer sequences are masked differently. The k-mer algorithm was used to divide aptamer sequences into nucleotide tokens using sliding windows. After tokenization, the tokens have dependencies for neighboring tokens. To eliminate these dependencies, we mask both the surrounding and individual tokens. For instance, if the tokenized sequence ACC, CCG, CGT, GTA, and TAC requires the third token to be masked, it would be replaced with [mask], creating the masked sequence ACC, [mask], [mask], [mask], and TAC. The pretraining module utilizes encoder representations to predict the original sequence from the masked sequence.

The second task, secondary structure prediction (SSP), was implemented in a manner similar to the first task. When the set of monomers or their order in a molecule changes, the structure of the molecule also changes because of the interactions between the monomers [43, 44]. As the binding sites of the molecules are determined by the monomers representing the sequences with the patterns of interactions between the monomers is crucial in identifying the structure of the molecules [45]. Because the SSP task is related to interactions between monomers, we pretrained our encoders for the SSP task to capture the patterns of these interactions. For this pretraining, we used protein sequences from the PDB and their secondary structures obtained by DSSP, as well as the 'bpRNA-1 m' RNA dataset with its secondary structures. The secondary structures of the molecules were tokenized according to token size of their sequences. For example, if a tokenized amino acid sequence reads as MSR, LDK, SK, …, LL, and QIV, and its associated secondary structure is SSSSHHHHH-…-TTSSS, then the secondary structure would be tokenized as SSS, SHHH, HH, …, TT, and SSS, as shown in Fig. 3 (bottom).

### Training AptaTrans using data augmentation techniques

While training the AptaTrans model, we employed several data augmentation techniques to alleviate any overfitting resulting from inadequate API data. One method involves expanding the training dataset by generating symmetrical aptamer molecules. Oligonucleotide aptamers have neither a distinct head nor tail, indicating that the symmetrical sequence of an aptamer can be considered the same molecule. For example, if an aptamer sequence is ACGAC and binds to the protein SVFSERT, its symmetrical sequence CAGCA is likely to bind to the same protein. Using this data augmentation approach, the size of the training dataset was effectively doubled.

### Experimental settings

AptaTrans was developed using PyTorch [46] and hyperparameters were determined through empirical results and consideration of available computing resources, as shown in Table 3. The model uses six-layer transformer encoders for protein and RNA sequences. These encoders utilize an input embedding size of 128 and integrate eight attention heads in their multi-head self-attention mechanism. The feed-forward layer of the encoders is designed to use a dropout rate of 0.1 and hidden dimension size of 512. During the training, we utilized the AdamW optimizer [47] with a learning rate of 1e-5.

**Table 3:** Optimal hyperparameters used for training the model

Input dimension	Number of heads	Dropout rate	Hidden dimension	Number of Layers	Optimizer	Learning rate
128	8	0.1	512	6	AdamW	1e-5

### Experiments and performance metrics

Our model was evaluated experimentally for two fundamental tasks: predicting API, which is a classification problem, and recommending candidate aptamer sequences, which is a generative simulation problem. For the binary classification task, we used six commonly used performance metrics: These performance metrics include the ROC-AUC, accuracy (ACC), Matthews correlation coefficient (MCC), sensitivity (Sn), specificity (Sp), and F1-score (F1), which are defined as follows:

7 $${\text{ROC - AUC = }}\int_{0}^{1} {{\text{ROC(}}x)dx}$$

8 $${\text{ACC = }}\frac{{\text{TP + TN}}}{{\text{TP + TN + FP + FN}}}$$

9 $${\text{MCC = }}\frac{{{\text{TP}} \times {\text{TN - FP}} \times {\text{FN}}}}{{({\text{TP + FP)(FP + FN)(TN + FP)(TN + FN)}}}}$$

10 $${\text{Sn = }}\frac{{{\text{TP}}}}{{\text{TP + FN}}}$$

11 $${\text{Sp = }}\frac{{{\text{TN}}}}{{\text{TN + FP}}}$$

12 $${\text{F1 = }}2 \times \frac{{{\text{TP}}}}{{\text{TP + FP + FN}}}$$

where TP, TN, FP, and FN denote the true positives, true negatives, false positives, and false negatives, respectively. The ROC curve illustrates how the binary classification performance varies according to its discrimination threshold. ${\text{ROC(}}x)$ represents the true positive rate, also known as the sensitivity, plotted against the false positive rate (1-specificity), for a given threshold $x$. The ROC-AUC is a crucial metric, particularly for the prediction of aptamer-protein Interaction. Predicting the interaction results in a binary outcome. Hence, the effectiveness of the model depends on its ability to accurately differentiate between binding and non-binding states. The ROC-AUC value measures the competence of the model in differentiating between these states across all potential thresholds. Accurately predicting the occurrence of binding (sensitivity) and when it does not occur (specificity) is crucial. False positives and negatives can lead to inefficient resource allocation in future research, such as in drug developments. An ideal model should have a high Sn and Sp, resulting in a high ROC-AUC value.

In the second task, which involves the generation and simulation, the results were obtained using the ZDOCK score acquired from the web-based ZDOCK Server. The ZDOCK Server is a web-based platform that offers access to the widely used bioinformatics tool ZDOCK for predicting protein–protein interactions and modeling protein complex structures. For the docking calculations, we submitted our protein and aptamer sequences to the ZDOCK Server. Following the docking simulation, the ZDOCK Server returned a score that estimated the binding affinity between the protein and aptamer. A higher ZDOCK score indicates a potentially strong interaction, further verifying the efficiency of the proposed aptamer sequences generated by Apta-MCTS using our AptaTrans model.

## Results and discussion

### Overview of the AptaTrans pipeline

**Fig. 4:**
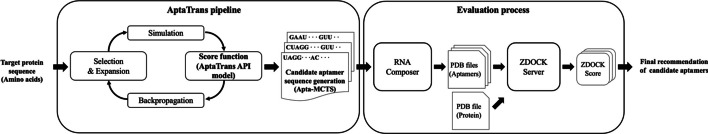
Candidate aptamer generation process and its analysis using the AptaTrnas pipeline (including Apta-MCTS), RNA Composer, and ZDOCK Server

We designed the AptaTrans pipeline and evaluated the process of generating and evaluating candidate aptamer sequences, as shown in Fig. 4. The AptaTrans pipeline consists of two main components: AptaTrans API prediction model and Apta-MCTS [25]. The AptaTrans API prediction model functions as an API classifier within Apta-MCTS, contributing to the generation of high-quality aptamer sequences in the pipeline. Apta-MCTS subsequently generates potential aptamer sequences that exhibit a high binding affinity for a target protein sequence, expressed as a series of amino acids. These potential aptamer sequences are returned in their nucleotide format. These sequences were evaluated using our evaluation process. The RNA Composer can convert RNA sequences, including aptamers, into PDB format files [48]. The PDB files were then submitted to the ZDOCK Server. On the ZDOCK Server, an interaction simulation occurs between the PDB files of the aptamer and protein sequences, resulting in a ZDOCK score. The score represents the predicted binding affinity. Thus, the calculated score indicates the predicted binding affinity between the protein and aptamer.

### API prediction performance

**Fig. 5:**
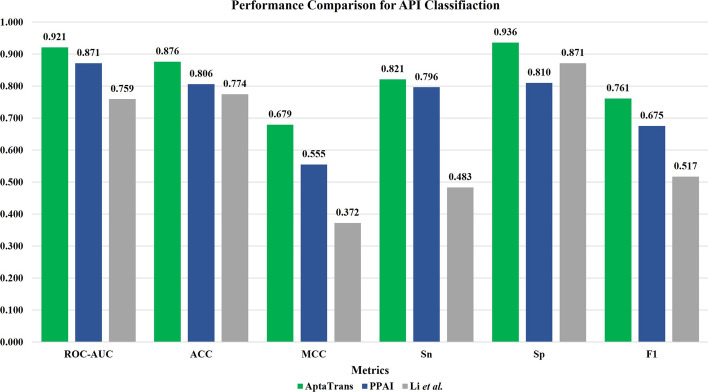
Performance comparison for API prediction in terms of six metrics: the ROC-AUC, accuracy (ACC), Matthews correlation coefficient (MCC), sensitivity (Sn), specificity (Sp), and F1-score. Our AptaTrans model was compared with PPAI [50] and Li et al.’s model [17]

We compared the performance of AptaTrans in predicting the binding of aptamer sequences and target proteins using two well-established API classifiers: PPAI [49] and Li et al.’s model [17]. The predictive models were evaluated using six aforementioned performance metrics. As shown in Fig. 5, AptaTrans outperformed the other models across all six metrics. The ROC-AUC score of our API prediction model was approximately 4.2% and 15.4% higher than those of PPAI and Li et al.’s model, respectively. AptaTrans achieved a better F1 than the other two methods, outperforming them by 6.9% and 22.7%, respectively. AptaTrans accurately identified true positives and negatives, as indicated by its high sensitivity (Sn) and specificity (Sp). In addition, it significantly outperformed the other models in terms of the MCC, with improvements of 9.8% and 28.1%, respectively. Overall, this performance comparison shows that AptaTrans predicts aptamer-protein interactions more accurately than other existing in silico methods.

### Ablation study for AptaTrans

**Fig. 6:**
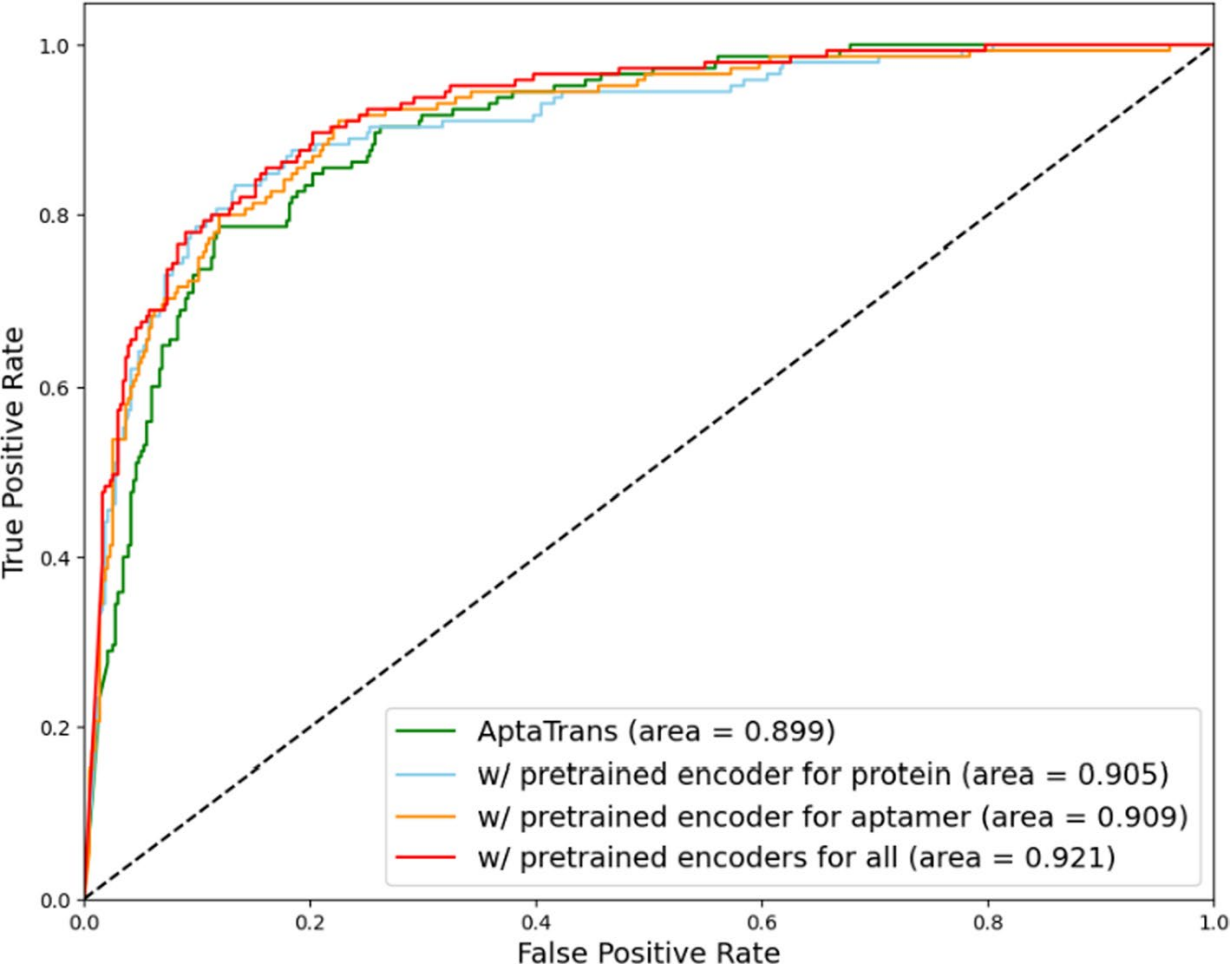
ROC curves comparing AptaTrans performance of baseline and pretraining setups

We conducted two ablation studies to evaluate the effects of the importance of pretraining and effects of model architecture components. Initially, we set up three different pretraining setups: one using a pretrained encoder for proteins, another using a pretrained encoder for aptamers, and the third integrating pretrained encoders for both. To assess the tangible benefits of pretraining, these setups were compared to the performance of the baseline, which is AptaTrans without any pretraining. The results are summarized in Table 4, showing that the baseline yielded an ROC-AUC of 0.899, an ACC of 0.857, an MCC of 0.639, and an F1 of 0.733. Notably, all pretraining setups outperformed this baseline. The most significant improvement was observed with the use of pretrained encoders for both proteins and aptamers: AptaTrans achieved an ROC-AUC of 0.921, an ACC of 0.876, an MCC of 0.679, and an F1 of 0.761. Our analysis, summarized in Table 4 and illustrated in Fig. 6, emphasizes the importance of pretraining in our model. Even configurations where only one encoder was pre-trained showed improved metrics in all areas. This iterative improvement confirms the crucial role of pretraining in enhancing the precision of the AptaTrans API prediction model, highlighting the value of this approach.

**Table 4:** Results of the pretraining ablation study

Model Setup	ROC-AUC	ACC	MCC	F1
AptaTrans	0.899	0.857	0.639	0.733
w/ pretrained encoder for protein	0.905	0.874	0.671	0.756
w/ pretrained encoder for aptamer	0.909	0.860	0.650	0.741
w/ pretrained encoders for all	**0.921**	**0.876**	**0.679**	**0.761**

Bold indicates the highest scores

In our secondary analysis, as described in Table 5, we evaluated the impact of different architectural components within the AptaTrans model. We conducted a comparative study without employing pre-trained encoders. Using the full proposed architecture in its original form, AptaTrans produced ROC-AUC metrics of 0.893, ACC of 0.857, MCC of 0.630, and F1 of 0.715. Removing the FCS mining component, which is crucial for tokenizing protein sequences, led to observable declines in accuracy, Matthews correlation coefficient, and F1 score. However, the ROC-AUC remained largely unchanged. This alteration negatively affected both precision and recall metrics. Performance declined for all metrics when a less complex encoder structure with only three layers and four heads of multi-head self-attention was chosen. The decrease in model performance highlights the effectiveness of our proposed multi-layer encoders in the efficient encapsulation of sequence contexts. Additionally, reducing the convolutional neural network (CNN) framework from a dense 17-block structure to a trimmed seven-block structure resulted in a significant decrease in model performance, underscoring the critical nature of careful feature extraction from interaction maps. Overall, our analyses clearly confirm the crucial contributions of selected model components to the effectiveness of AptaTrans, in particular FCS mining, sophisticated encoders, and complex CNN blocks.

**Table 5:** Results of the ablation study for model architecture w/o pretraining

Model setup	ROC-AUC	ACC	MCC	F1
AptaTrans	**0.899**	**0.857**	**0.639**	**0.733**
w/o FCS mining	0.893	0.851	0.627	0.708
w/ simple Encoders	0.879	0.850	0.583	0.677
w/ shallow CNN	0.832	0.819	0.537	0.658

Bold indicates the highest scores

### Candidate aptamer sequence recommendation

We have developed the AptaTrans pipeline, which combines AptaTrans with Apta-MCTS [25]. This pipeline is designed to generate candidate aptamer sequences for a target protein. Apta-MCTS has two phases: searching for potential aptamers with a high binding affinity propensity, based on Monte Carlo tree search (MCTS)-based sampling, and predicting the binding scores of the candidate aptamers for the given target protein using the API prediction model. In order to improve the accuracy, we replaced the original Apta-MCTS API prediction model with the AptaTrans API prediction model.

**Fig. 7:**
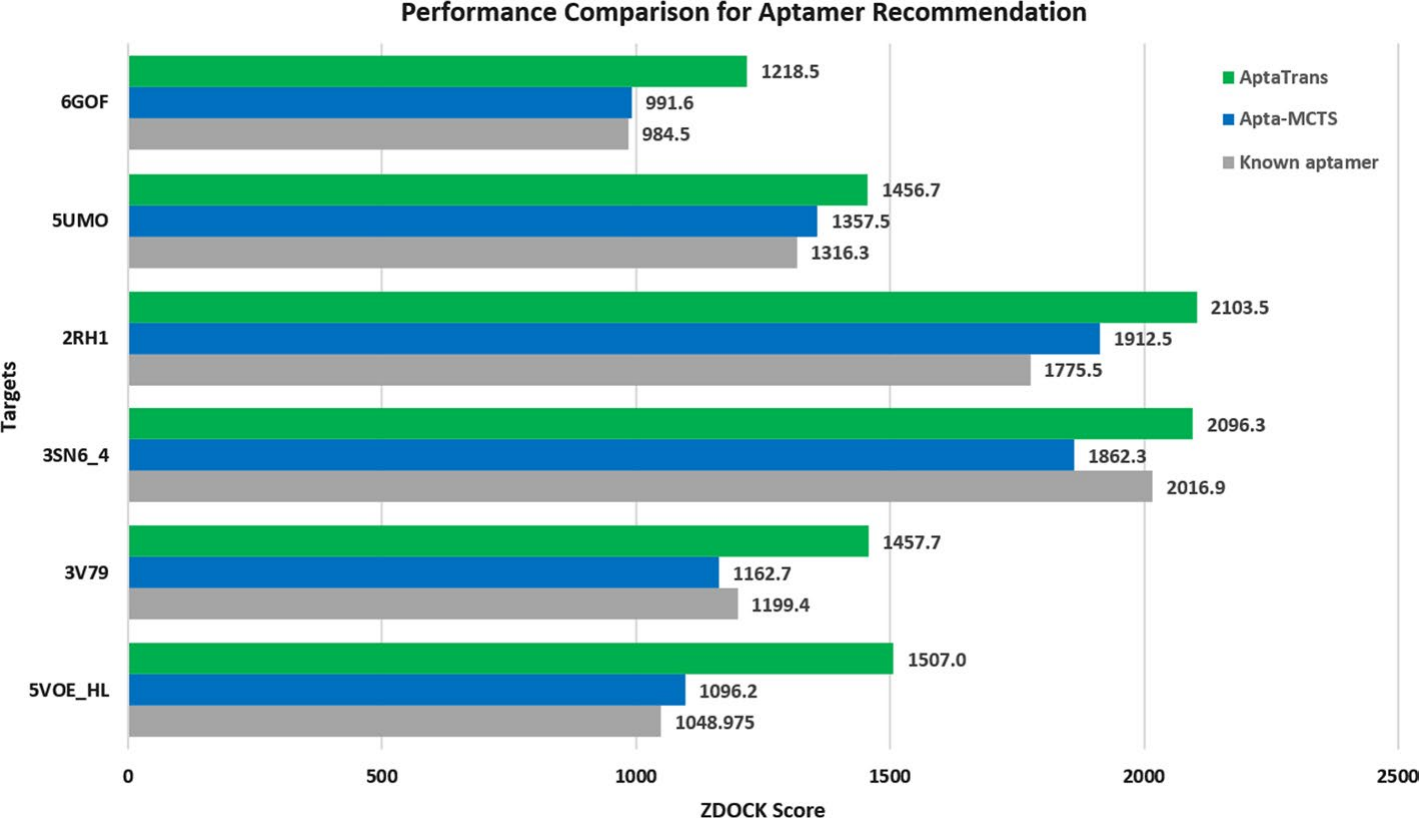
Performance comparison for aptamer sequence recommendation

We assessed the binding positions and ZDOCK scores of the candidate aptamers generated by our AptaTrans pipeline for six proteins: 6GOF, 5UMO, 2RH1, 3SN6_4, 3V79, and 5VOE_HL. These six proteins have already been investigated using well-known aptamers. We compared the ZDOCK scores of these candidate aptamers with known and other candidate aptamers generated by the original Apta-MCTS, which was already superior to the known aptamers. Figure 7 shows the ZDOCK scores of the candidate aptamer sequences for the six target proteins obtained using the ZDOCK docking server. According to the ZDOCK scores, the AptaTrans pipeline demonstrated higher scores than previous results for Apta-MCTS and the known aptamers as illustrated in Fig. 7.

### Analysis with known aptamer and candidate aptamers

We performed a comparative analysis of a known aptamer and AptaTrans found candidate aptamers generated using the AptaTrans pipeline in the aspect of quantity and quality. In quantity aspect, we compare ZDOCK score between the protein interaction of the known aptamer and top ranked AptaTrans candidate aptamers. In Fig. @10 we compare the ZDOCK score of the known aptamer for 6GOF and 3SN6_4 between top 2 aptamers which found by AptaTrans pipeline. Results shows that the ZDOCK score of the known aptamer for 6GOF is 1016.107 whereas top ranked AptaTrans candidate aptamers score are 1249.581 and 1387.074 respectively, which 370.967 points higher than the known aptamer. For the ZDOCK score of 3SN6_4, the known aptamer scores 2053.519 while top ranked AptaTrans candidate aptamers score are 2139.379 and 2271.218 respectively, which also increased the points by 217.699.

**Fig. 8:**
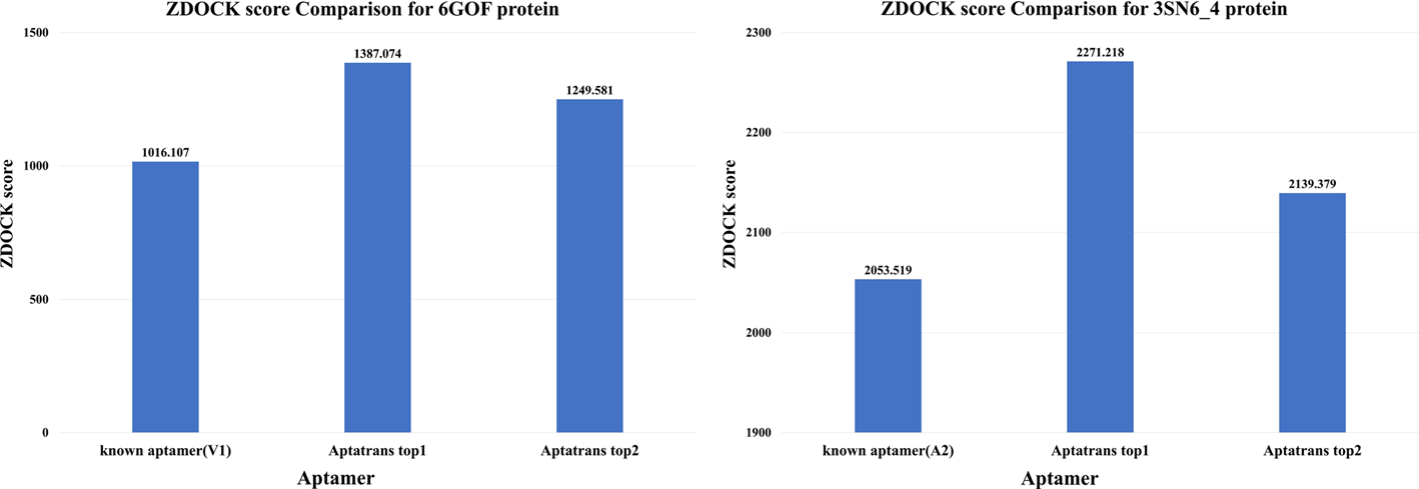
ZDOCK score comparisons between known aptamers and candidate aptamers for proteins 6GOF and 3NS6

For quality analysis, we used PyMOL [50] to visualize the aptamer-protein complexes formed. Figure 8A shows the complexes formed by the known aptamers and the candidate aptamers upon binding to the 6GOF protein. In contrast, Fig. 8B illustrates known and candidate aptamers upon binding to the 3SN6_4 protein. The upper figures in Fig. 8 show the binding configuration with the position of the protein (in green) and the aptamer (in red). Conversely, the lower figures highlight the crucial primary binding sites necessary for protein and aptamer interaction. Interestingly, both the known and our candidate aptamers show affinity for similar binding sites on the 3SN6_4 and 6GOF proteins, as shown in Fig. 8.

**Fig. 9:**
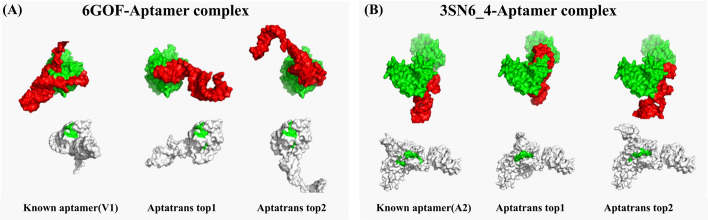
Visualization of protein complex with known aptamers and top two candidate aptamers generated by AptaTrans pipeline for proteins 6GOF and 3SN6_4

**Fig. 10:**
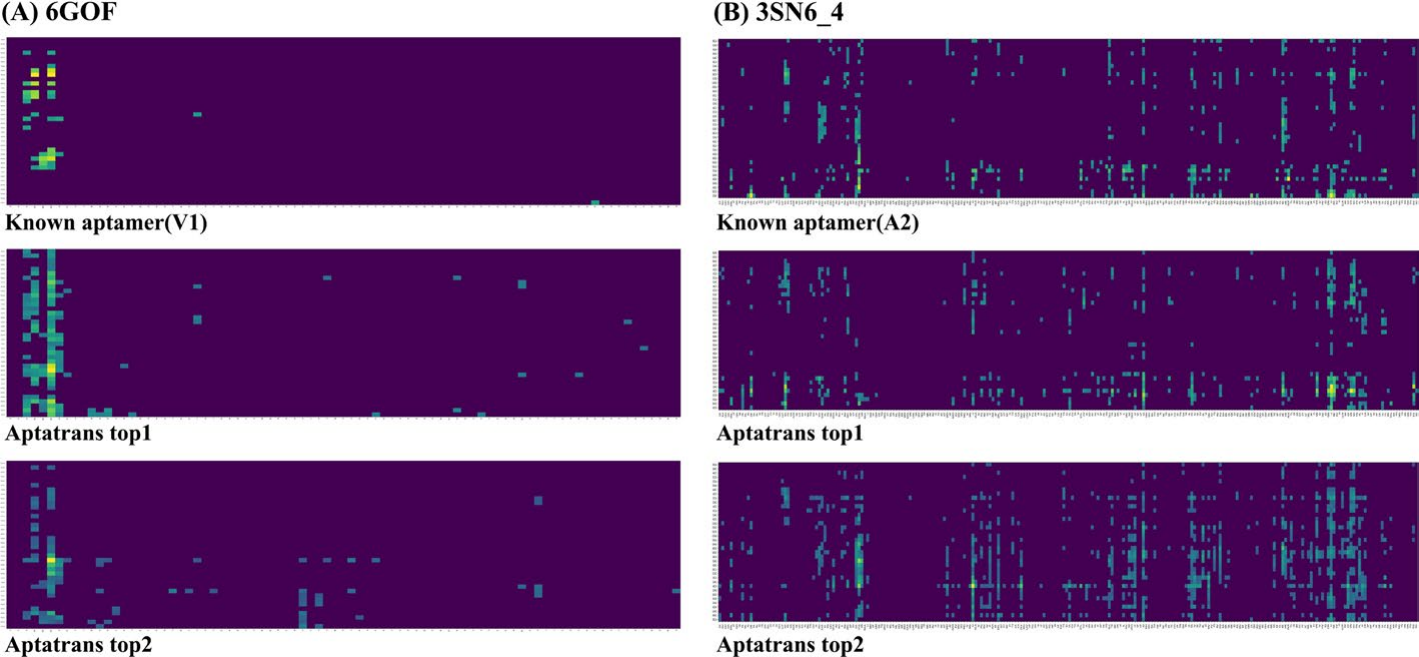
Visualization of AptaTrans interaction maps for aptamer sequences and target proteins. X-axis and y-axis indicate protein and aptamer sequence tokens respectively. The tokens that show higher values than a selected threshold value in the interaction map are marked in bright color. This illustrates that that both known and candidate aptamer sequences exhibit high values in similar regions of the protein sequence

Furthermore, we conducted additional analysis about the relationship between aptamer and protein sequences. For this analysis, we obtained the interaction maps of aptamer and protein sequences using AtpaTrans for predicting API. Fig. @10 shows the interaction maps of the known aptamer and the two candidate aptamers with 6GOF and 3SN6_4 proteins. As shown in Fig. 2A, the interaction maps are calculated through dot product with contextualized embeddings of both aptamer and protein sequences using transformer encoders. These embeddings represent the sequence-related knowledge. For clarity of visualization, we set the threshold values and mark sequence tokens that show higher values than the selected threshold. As shown in Fig. 10, the interaction map illustrates that both known and candidate aptamer sequences exhibit high values in similar regions of the protein sequence, similar to what is shown in Fig. 9. We identified that the interaction maps reveal notable interaction points between the aptamer and target protein [37] (Fig. 10).

### Motif analysis of aptamer sequences

**Fig. 11:**
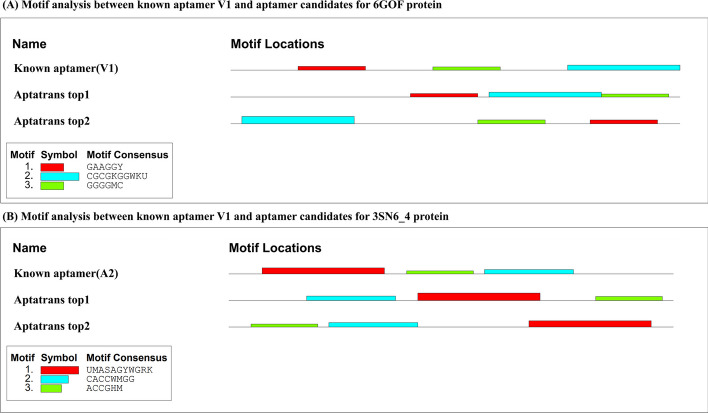
Motif analysis between known aptamer and candidate aptamers for two proteins, **A** 6GOF and **B** 3NS6

We visualized and analyzed the motifs of the candidate aptamer sequences generated by our AptaTrans pipeline and known aptamers. Motifs between candidate and known aptamers were identified using MEME [51]. Figure 11A shows the MEME motif results between the known aptamer (V1) and top-two candidate aptamers for protein 6GOF, and Fig. 11B shows those for protein 3SN6_4. Although the motif locations differ, some major motifs are present in both the known aptamers and top-two candidate aptamers in both proteins. This suggests that the binding sites are similar between the known aptamers and our candidate aptamers. This indicated that the candidate aptamers generated by AptaTrans are highly likely to bind to the target proteins.

### Comparison of aptamers from SELEX and AptaTrans

We conducted an experiment to compare DNA aptamer PS202 aptamer from SELEX with aptamers generated using the proposed AptaTrans pipeline. The PS202 aptamer was derived from SELEX experiments targeting the protein glutamate carboxypeptidase II (GCPII), also known as the prostate-specific membrane antigen [52]. From the pool of candidate sequences produced by AptaTrans, we selected two aptamers based on their superior ZDOCK scores for the GCPII protein.

**Fig. 12:**
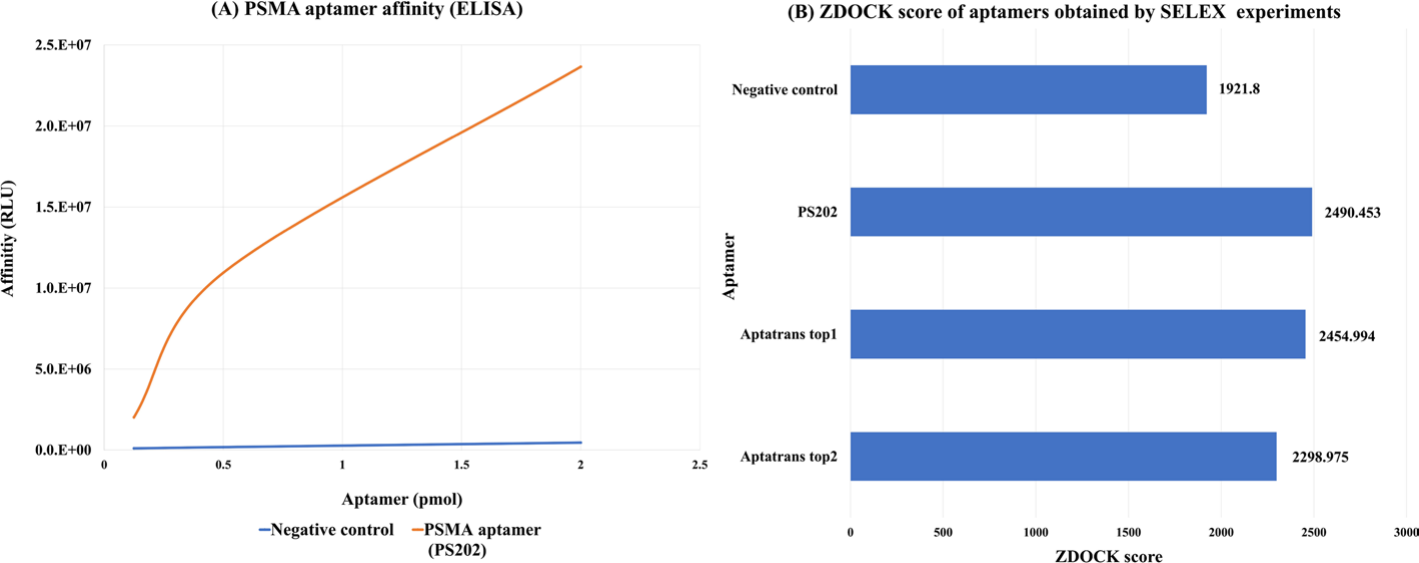
ELISA and ZDOCK simulation results for protein ‘glutamate carboxypeptidase2.’

First, we assessed the effectiveness of the PS202 aptamer using enzyme-linked immunosorbent assay (ELISA). This well-established method is commonly used to measure proteins, antibodies, antigens, and other biomolecules [53]. Since our aptamer candidates generated by AptaTrans are RNA aptamers, we converted the PS202 aptamer to its corresponding RNA sequence in Fig. 12. Figure 12A still shows a significant improvement in the binding affinity of the PS202 aptamer to its target protein with an increase in the aptamer concentration. This observation highlights the superior performance of the PS202 aptamer compared with that of the negative control. Next, we conducted ZDOCK docking simulations to obtain the ZDOCK scores of the negative control, PS202, and the candidate aptamers. The resulting data, as shown in Fig. 12B, indicate that although the PS202 aptamer in RNA sequence had the highest ZDOCK score, the scores of the top two candidate aptamers were considerably close. This shows that the RNA aptamer candidate sequences generated by AptaTrans can be utilized in the development of DNA or modified DNA aptamers. These findings emphasize the potential of the AptaTrans pipeline in reducing the time and financial costs typically associated with SELEX experiments.

**Fig. 13:**
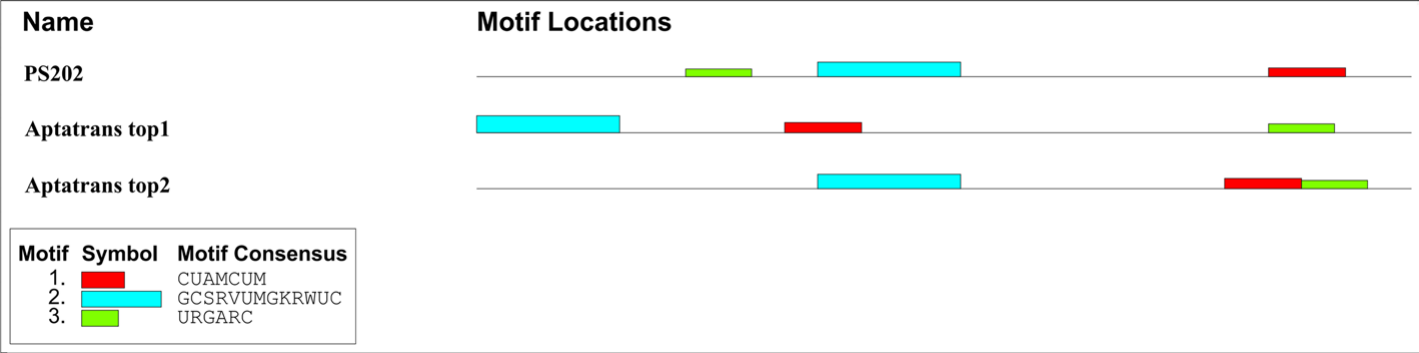
Motif analysis between the PS202 aptamer and two candidate aptamers for the GCPII proteins

Next, we conducted a thorough motif analysis of the aptamer sequences obtained from the AptaTrans pipeline in comparison with PS202. The MEME motif analysis compared PS202 with the two most prominent candidate aptamers targeting the GCPII protein, as shown in Fig. 13. Similar to the discoveries in Fig. 11, it is important to note that significant motifs persistently appeared in both PS202 and the top two candidate aptamers across the proteins under consideration, despite differences in motif spatial location.

## Conclusion

Identifying aptamer sequences that effectively bind to target proteins is critical in both biological research and drug discovery. In this paper, we introduce the AptaTrans pipeline, an integration of a deep learning framework designed to predict aptamer-target protein interaction and Apta-MCTS, which generates candidate aptamers. The model for predicting the API, named AptaTrans, leverages the relationship between the aptamer and the subsequences of the protein to predict the API. In particular, this model employs pretrained encoders that utilize advanced techniques to predict masked tokens and secondary structures. Our results demonstrate the impressive performance of AptaTrans and highlight the benefits of pre-training in enhancing the model's ability to understand sequences. Further validation of the aptamers generated by the AptaTrans pipeline was conducted using RNA Composer and the ZDOCK server. Notably, the aptamers generated by our pipeline outperformed both their Apta-MCTS counterparts and known aptamers when evaluated by ZDOCK. These results suggest that the AptaTrans pipeline is superior to existing methods in terms of its superior performance capabilities. In addition, we evaluated the quality of our aptamers using binding position visualization with PyMOL and motif analysis with MEME. Both binding positions and motifs of our aptamer sequences showed significant similarities to known aptamers. When compared to known aptamers identified through SELEX experiments, our candidate aptamers show superior quality, with ZDOCK scores that are comparable to or higher than those of existing aptamers. Although our pipeline searches for candidate aptamers by considering both sequence and secondary structure information, it still has some limitations. One significant drawback involves the inability to confirm the affinity of AptaTrans-generated candidates through biological experiments. While we also obtained satisfactory results by conducting experiments assuming the same environmental factors, external environmental factors such as temperature, acidity (pH) and ionic strength could affect the binding. We expect that considering these additional factors could lead to improve our aptamer generation pipeline. As part of our future work and validation process, we aim to extend our API prediction model of which the scores can be utilized to quantify the binding affinity if the concentration of the aptamer-protein complex can be measured using binding assays. Our pipeline could be also validated for its potential use in comprehending the specificity of aptamer-protein interactions through fluorescence-based assays or mass spectrometry. In addition, the tertiary structure prediction of proteins performed by AlphaFold could lead to the precise prediction of aptamer-protein interactions. AptaTrans has shown outstanding performance on our benchmark RNA aptamer-protein dataset. These achievements may open the door to establishing unique and novel interactions between aptamers and specific targets. We believe that our AptaTrans pipeline (in silico) not only reduce the time and cost required by the SELEX (in vitro) method, but also provide valuable biological insights to researchers in the aptamer field and lead to significant progress in drug discovery research.

## Data Availability

The API prediction dataset, based on experimental aptamer-protein complex data, was derived from Li et al. [17]. The datasets used for pretraining the encoders for proteins and aptamers are publicly available at the Protein Data Bank (PDB) [32] and bpRNA-1 m (commonly referred to as bpRNA) [33], respectively. The source code and benchmark dataset for AptaTrans are available at https://github.com/pnumlb/AptaTrans.
